# Supplementary material for: An Extensive Metabolomics Workflow to Discover Cardiotoxin-Induced Molecular Perturbations in Microtissues
Source: Metabolites. 2021 Sep 21;11(9):644. doi: 10.3390/metabo11090644 (PMC8470535; doi:10.3390/metabo11090644)
Supplement: Supplementary file 1 [file metabolites-11-00644-s001.zip › metabolites-1346014-supplementary/metabolites-1346014-supplementary-revised-TJB/Supplementary_Materials.pdf]

Supplementary Information

An extensive metabolomics workflow to discover cardiotoxin-induced molecular perturbations in microtissues

Tara J. Bowen [1], Andrew R. Hall [2], Gavin R. Lloyd [3], Ralf J.M. Weber [1,3], Amanda Wilson [4], Amy Pointon [2] and Mark R. Viant [1,3,*]

[1] School of Biosciences, University of Birmingham, Edgbaston, Birmingham B15 2TT, UK; tjb413@student.bham.ac.uk (T.J.B.); r.j.weber@bham.ac.uk (R.J.M.W.)

[2] Functional and Mechanistic Safety, Clinical Pharmacology and Safety Sciences, R&D, AstraZeneca, Cambridge, CB4 0WG, UK; andrew.hall@astrazeneca.com (A.R.H.); amy.pointon@astrazeneca.com (A.P.)

[3] Phenome Centre Birmingham, University of Birmingham, Edgbaston, Birmingham B15 2TT, UK; g.r.lloyd@bham.ac.uk

[4] Clinical Pharmacology and Quantitative Pharmacology, Clinical Pharmacology and Safety Sciencies, BioPharmaceuticals R&D, AstraZeneca, Cambridge, CB4 0WG, UK; amanda.wilson@astrazeneca.com (A.W.)

* Correspondence: m.viant@bham.ac.uk

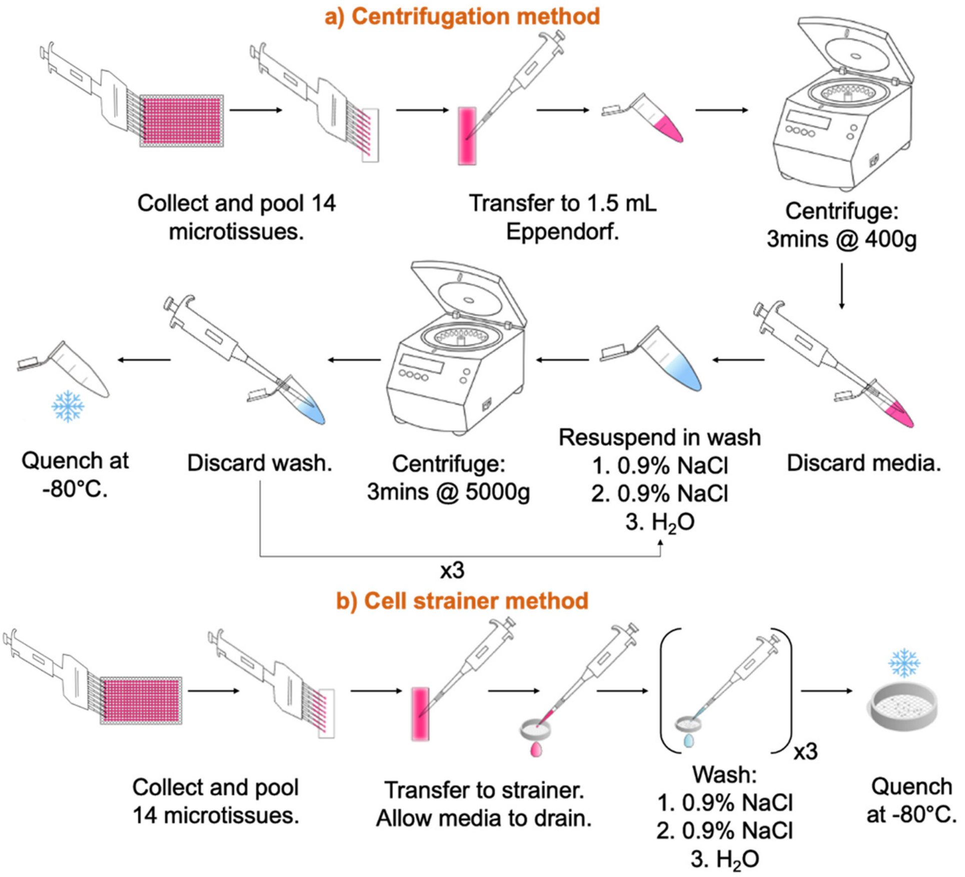

Figure S1. Graphical summary of two approaches for sampling cardiac microtissues, incorporating isolation from culture medium, washing and quenching of metabolism, are displayed: **a** centrifugation-based method and **b** filtration-based method employing cell strainers. Both methods begin by collecting the microtissues and their culture medium from the culturing 384-well microplate and pooling into a reservoir. For isolation from the media by centrifugation, **a**, the pooled material (media and microtissues) is dispensed into a microcentrifuge tube, then centrifuged to pellet the cellular material, allowing the media/supernatant to be discarded. Washing is achieved by resuspending the cell pellet with saline (0.9% NaCl) or water, and repetition of the centrifuge/discard supernatant cycle until the cellular material has been washed twice with saline and once with water. For isolation of the microtissues from the media by filtration, **b**, the pooled material (media and microtissues) is dispensed onto a cell strainer. The media is allowed to drain through the filter, whilst the microtissues are retained on the surface. Subsequent washing is achieved by addition of either saline or water to the cell strainer, which also drains to waste, diluting and removing any residual media. The final step for both approaches is to quench metabolism by flash-freezing the samples, contained, **a**, within a microcentrifuge tube or, **b**, on the filter mesh of a cell strainer, in dry ice/ethanol bath (*ca.* -78°C) and storing at -80°C until analysis.

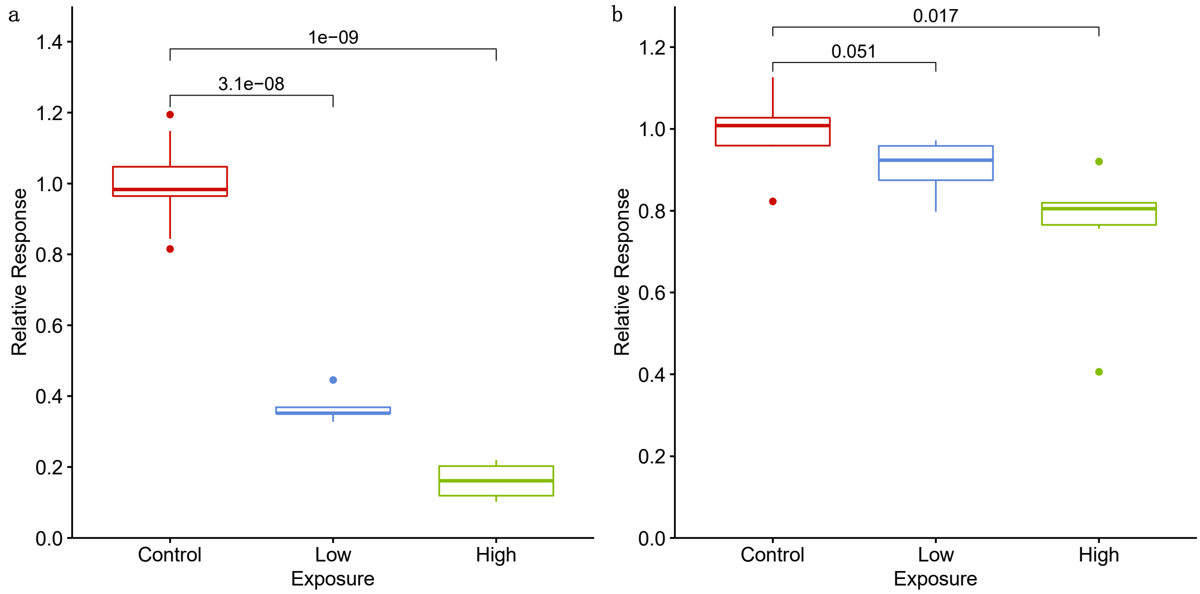

Figure S2. Box plots showing the relative change to **a** endoplasmic reticulum integrity and **b** mitochondrial membrane potential of cardiac microtissues exposed to either low (1.1 μM) or high (3.5 μM) concentration of sunitinib, compared to controls, as measured by high content biology imaging assays. p-values calculated by two-sided t-tests are displayed.

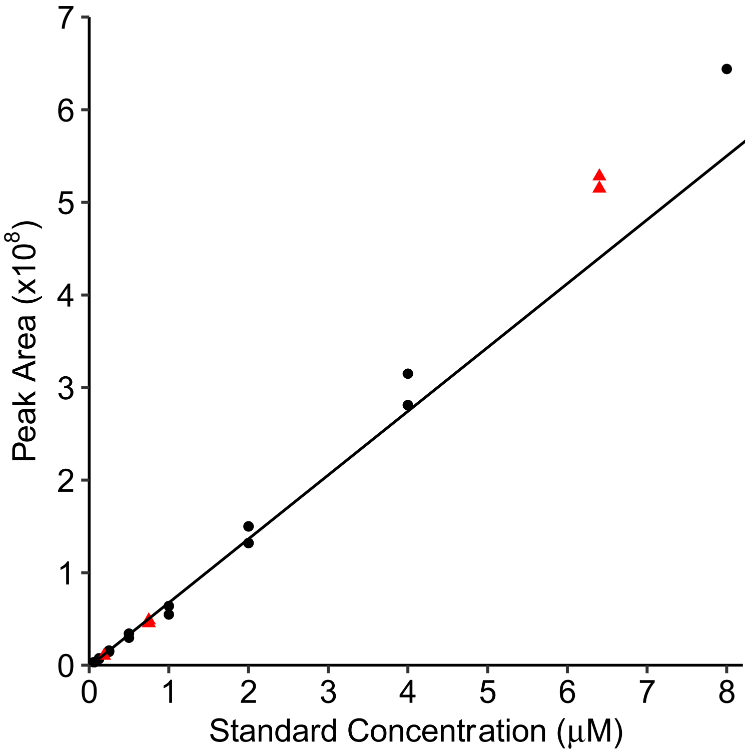

Figure S3. External calibration curve for sunitinib. Black points (●) show measured peak areas of accepted external calibration standard samples and red points (▲) show peak areas of accepted toxicokinetic quality control samples (TK QCs) (acceptance criteria for calibration standards and TK QCs are described in Section 3.5.5). A $1/x^2$ linear curve with formula $y = 6.90 \times 10^7 x - 1.15 \times 10^6$ was fitted to the external calibration standard data using TraceFinder (Thermo Fisher). The calibration curve fits the data with R^2 = 0.99.

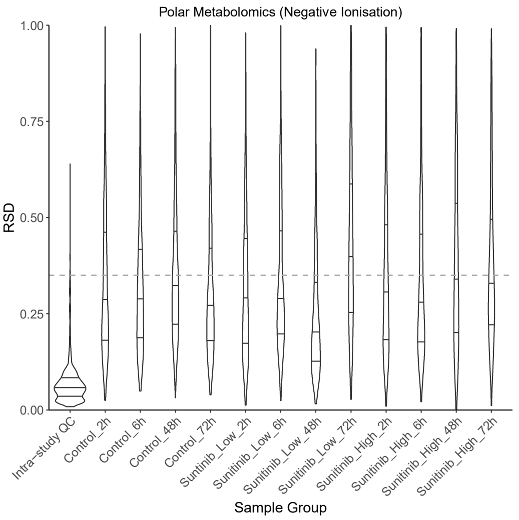

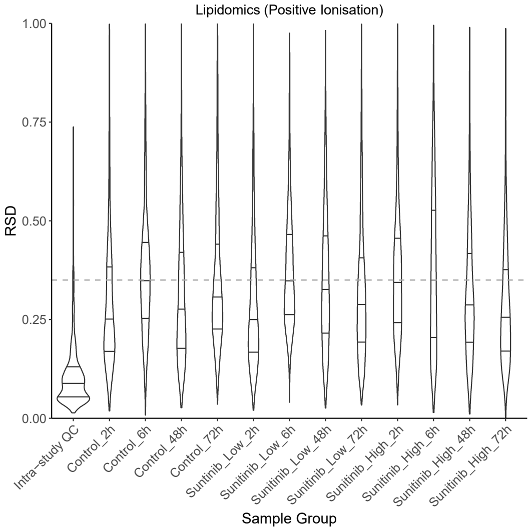

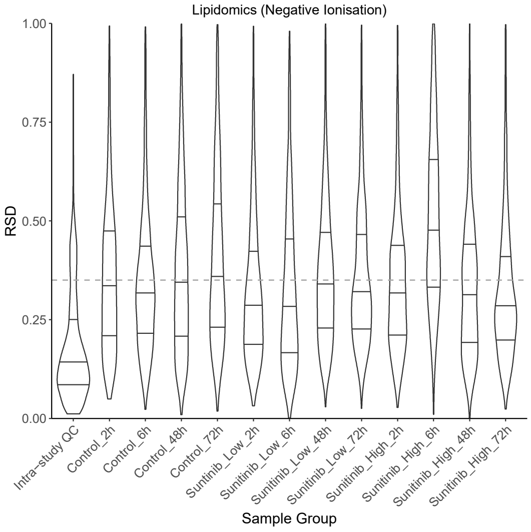

Figures S4 – S6. Violin plots displaying the distribution of feature intensity RSDs measured per sample group for nESI-DIMS negative metabolomics, positive lipidomics and negative lipidomics assays, respectively.

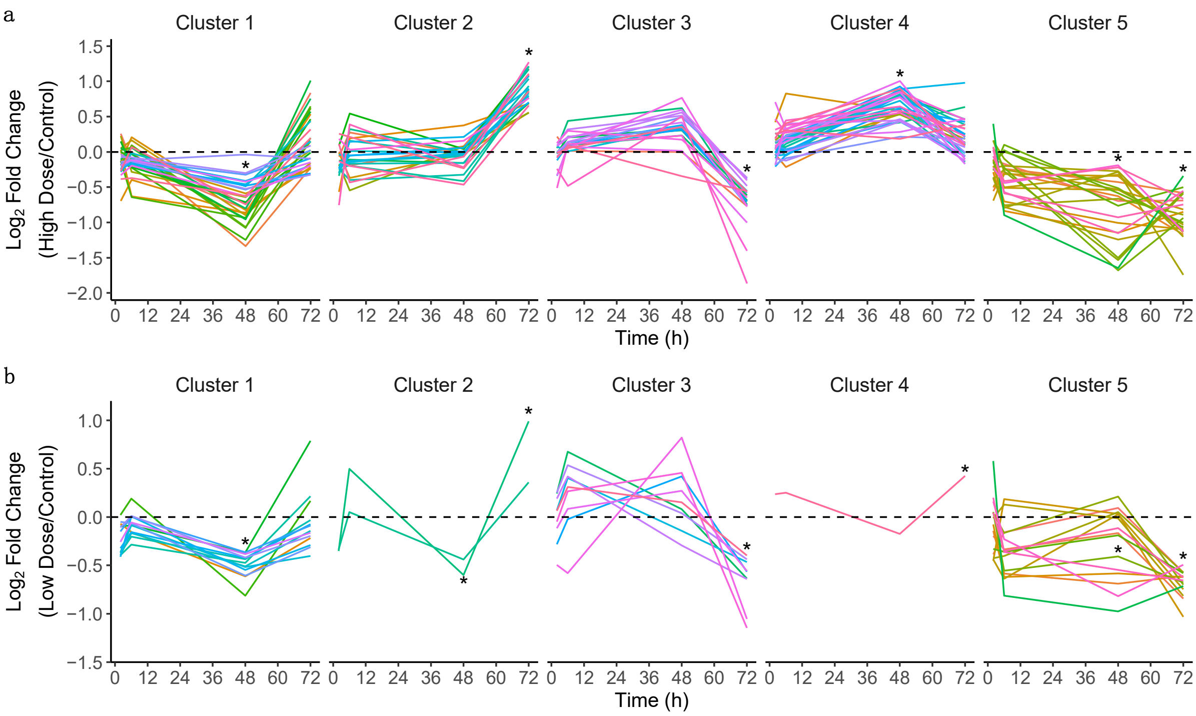

Figure S7. a Clusters of temporal response (log2 fold change, treated compared to control, at 2, 6, 48 and 72 h) of annotated lipid features showing significant change (p-value < 0.05, 1-way ANOVA with Tukey's HSD) in relative levels in microtissues exposed to high concentration of sunitinib were calculated by k-means cluster analysis (k = 5, selected using elbow method). These response patterns correspond to a reduction at 48 h but with recovery to normal levels by 72 h, increase in levels at 72 h, reduction at 72 h, increase at 48 h but with recovery to normal levels by 72 h and a reduction at 48 and 72 h for clusters 1, 2, 3, 4, and 5, respectively. **b** Analysis of the same lipids following exposure to low concentration sunitinib reveals comparable time-dependent significant responses of smaller magnitude for 12 of 30, 9 of 21, and 15 of 24 lipids belonging to clusters 1, 3 and 5, respectively, revealing a dose-dependency of these temporal patterns. Only one of the lipids aligning to cluster 4 displayed a significant response to low exposure, with the significantly different levels occurring at 72 h. Furthermore, only two of the lipids aligning to cluster 2 significantly responded to the low concentration of sunitinib, one at 48 h, and one at 72 h. * p < 0.05 at specified time point for the majority of features shown in the cluster as measured by one-way ANOVA with Tukey's HSD post hoc test.

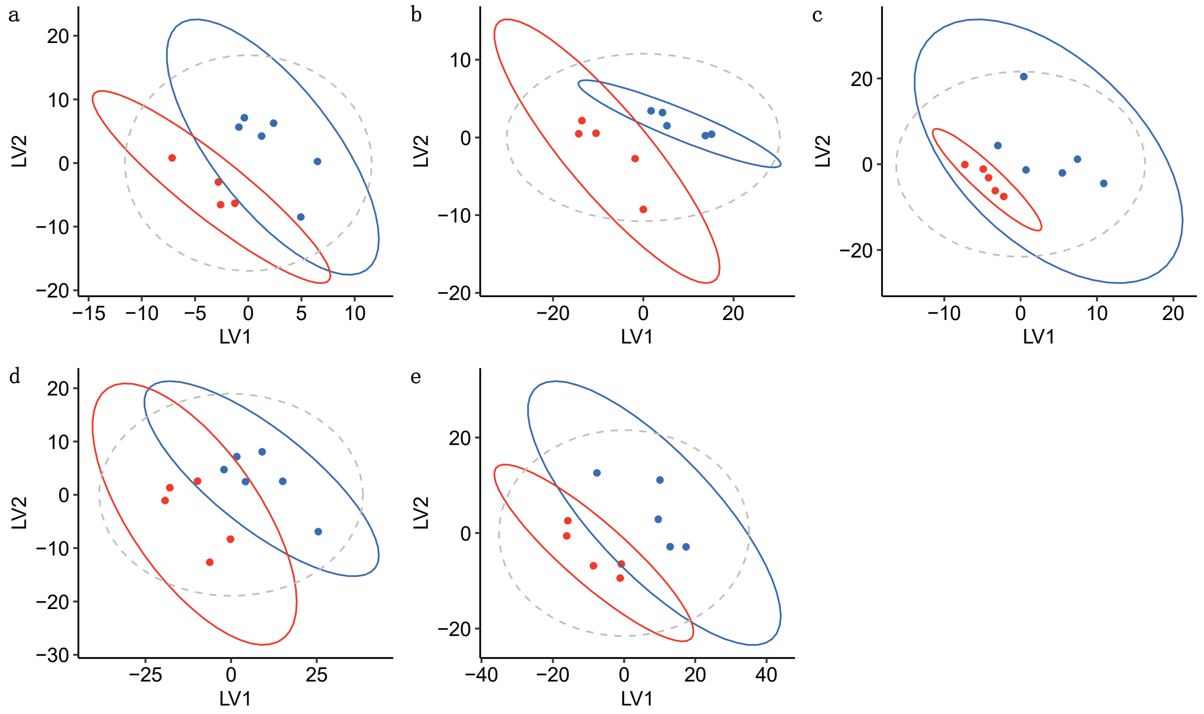

Figure S8. Scores plots of PLS-DA models (latent variables 1 and 2) discriminating between the negative ion metabolome, of microtissues exposed to a low concentration (1.1 μM) of sunitinib (red) and control microtissues (blue) at **a** 2 h, **b** 6 h and **c** 48 h after initial exposure. Scores plots of PLS-DA models (latent variables 1 and 2) which discriminate between the negative ion lipidome of microtissues exposed to a high concentration (3.5 μM) of sunitinib (red) and control microtissues (blue) at **d** 48 hrs and **e** 72 h are also displayed. Solid lines show 95% confidence interval of each group. The dashed grey line shows the 95% confidence interval of all samples.

Table S2. Performance metrics for PLS-DA models. The mean R^2 ± standard deviation as calculated by 10-fold cross-validation (using the leave-one-out method), Q^2, balanced error rate (BER) and BER p-value, as calculated using the formula $P = \frac{1+\#(NMC_p \leq NMC)}{N}$, where NMC is the number of misclassifications, $\#(NMC_p \leq NMC)$ is the number of elements in the null distribution which are smaller or equal to the NMC for the original dataset and N is the number of permutations (100), are reported for each PLS-DA model.

Metabolomics assay	Comparison	Time (h)	R^2 ± Std Dev	Q^2	BER	BER p-value
Polar Negative	Low Dose vs Control	2	0.94 ± 0.04	0.51	0.083	0.02
Polar Negative	Low Dose vs Control	6	0.94 ± 0.02	0.65	0.1	0.03
Polar Negative	Low Dose vs Control	48	0.91 ± 0.02	0.70	0	0.01
Non-Polar Negative	High Dose vs Control	48	0.95 ± 0.02	0.64	0.18	0.05
Non-Polar Negative	High Dose vs Control	72	0.96 ± 0.01	0.51	0.1	0.02

Table S4. Results of over-representation pathway analysis of metabolites significantly perturbed by sunitinib exposure in cardiac microtissues, performed using MetaboAnalyst [50]. The number of metabolites (putatively annotated) that were significantly perturbed by sunitinib exposure which align to each KEGG metabolic pathway [37] (Number of hits) and the total number of metabolites in each KEGG metabolic pathway (Total number of metabolites in pathway) are reported. The expected number of hits refers to the number of metabolites within a given pathway expected to be within the list of significantly changing metabolites in the case of no enrichment (depends on number of significantly changing metabolites and number of metabolites in pathway). The fold enrichment of significantly changing metabolites (putative annotations) aligning to each KEGG metabolic pathway was calculated as the ratio of number of hits to expected number of hits, and the significance assessed by a hypergeometric test (p-value, q-value with FDR correction). The top four enriched pathways are displayed.

Pathway	Total number of metabolites in pathway	Expected number of hits	Number of hits	Fold Enrichment	p-value	q-value (FDR correction)
Purine metabolism	65	1.26	7	5.6	0.0002	0.0131
Pyrimidine metabolism	39	0.75	3	4.0	0.0374	1.000
Lysine degradation	25	0.48	1	2.1	0.389	1.000
Galactose metabolism	27	0.52	1	1.9	0.413	1.000

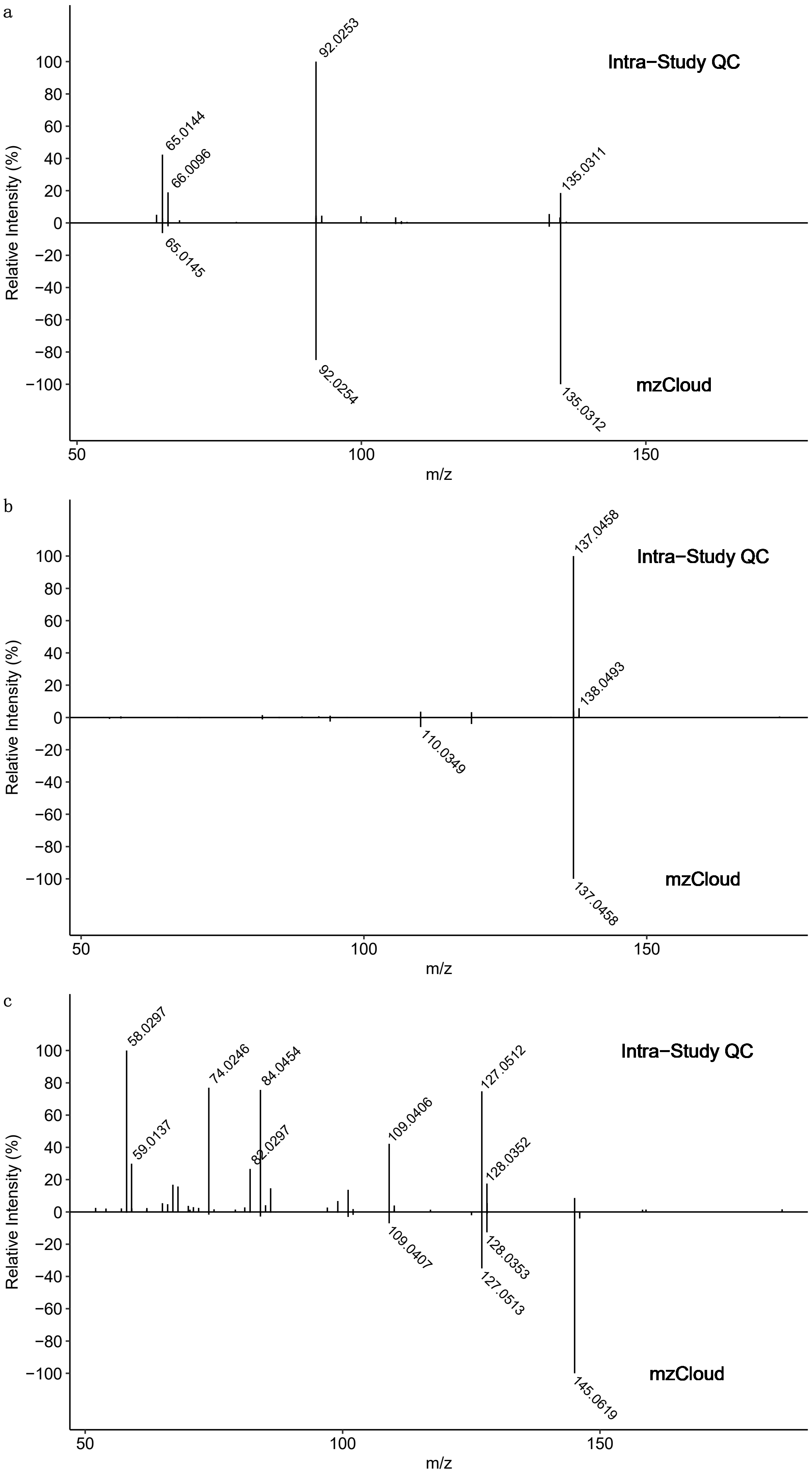
a
Intra−Study QC
mzCloud
Relative Intensity (%)
m/z
92.0253
65.0144
66.0096
135.0311
65.0145
92.0254
135.0312
b
Intra−Study QC
mzCloud
Relative Intensity (%)
m/z
137.0458
138.0493
110.0349
137.0458
c
Intra−Study QC
mzCloud
Relative Intensity (%)
m/z
58.0297
74.0246
84.0454
127.0512
59.0137
82.0297
109.0406
128.0352
109.0407
128.0353
127.0513
145.0619

Figure S9. Comparison of measured MS/MS fragmentation spectra for each annotated feature (top) and reference spectra for the corresponding metabolite from the mzCloud library (www.mzcloud.org; bottom). Spectral match scores of 86.1%, 86.4% and 78.8%, were calculated for **a** hypoxanthine, **b** inosine and **c** L-glutamine, respectively, using the HighChem HighRes algorithm in Compound Discoverer (Thermo Scientific).

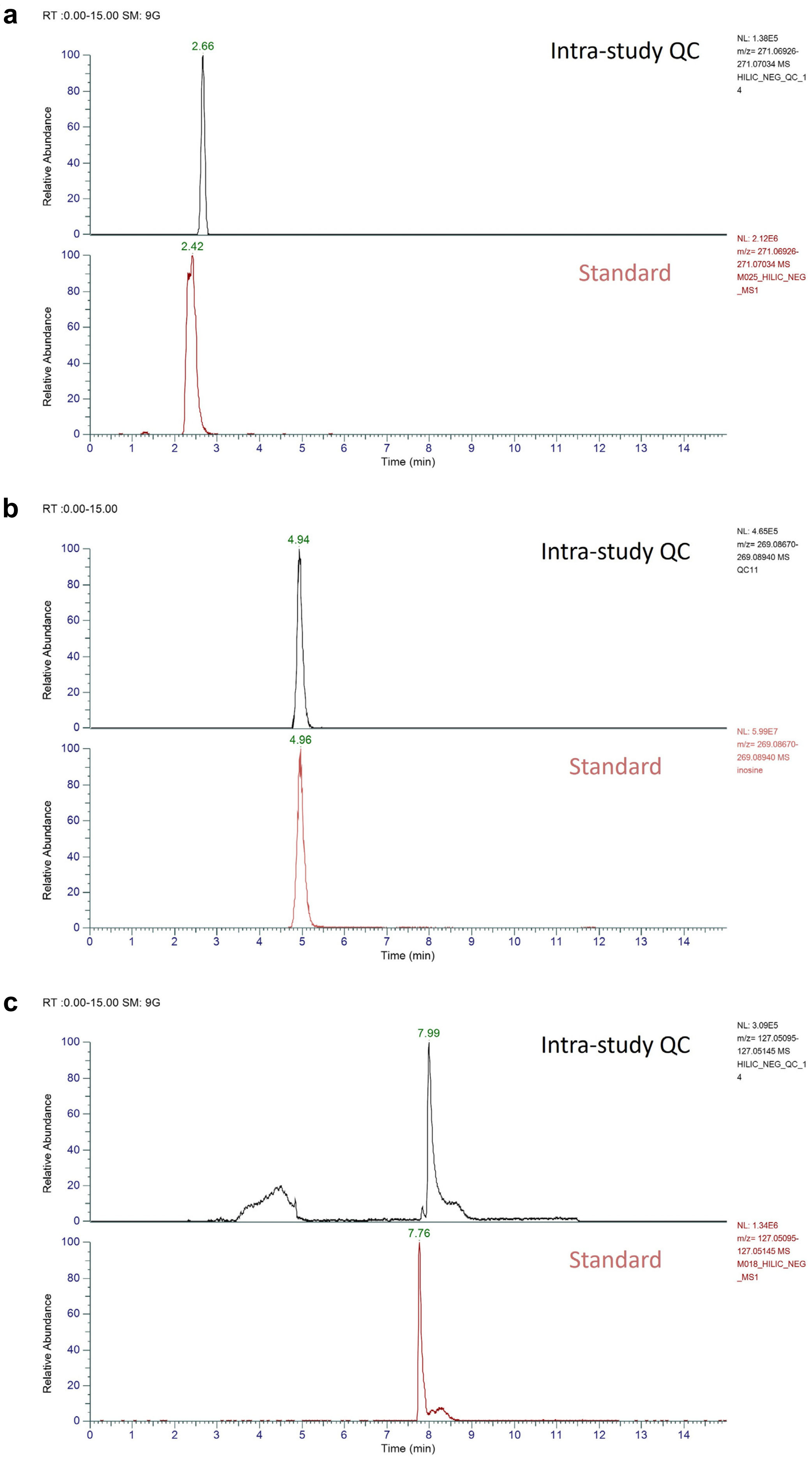
a
RT :0.00-15.00 SM: 9G
Intra-study QC
NL: 1.38E5
m/z= 271.06926-271.07034 MS
HILIC_NEG_QC_14
2.66
Standard
NL: 2.12E6
m/z= 271.06926-271.07034 MS
M025_HILIC_NEG_MS1
2.42
Relative Abundance
Time (min)
b
RT :0.00-15.00
Intra-study QC
NL: 4.65E5
m/z= 269.08670-269.08940 MS
QC11
4.94
Standard
NL: 5.99E7
m/z= 269.08670-269.08940 MS
inosine
4.96
Relative Abundance
Time (min)
c
RT :0.00-15.00 SM: 9G
Intra-study QC
NL: 3.09E5
m/z= 127.05095-127.05145 MS
HILIC_NEG_QC_14
7.99
Standard
NL: 1.34E6
m/z= 127.05095-127.05145 MS
M018_HILIC_NEG_MS1
7.76
Relative Abundance
Time (min)

Figure S10. Extracted ion chromatograms (EICs) showing matching retention times (± 30 s tolerance) between each annotated feature, as measured in an intra-study QC sample (top EIC, labelled “intra-study QC”), and the corresponding reference standard (bottom EIC, labelled “standard”). **a** hypoxanthine $(2M - H]^-$, *m/z* 271.0697), **b** inosine ($[M + H]^+$, *m/z* 269.0880) and **c** L-glutamine ($[M – H - H_2O]^-$, *m/z* 127.0513).

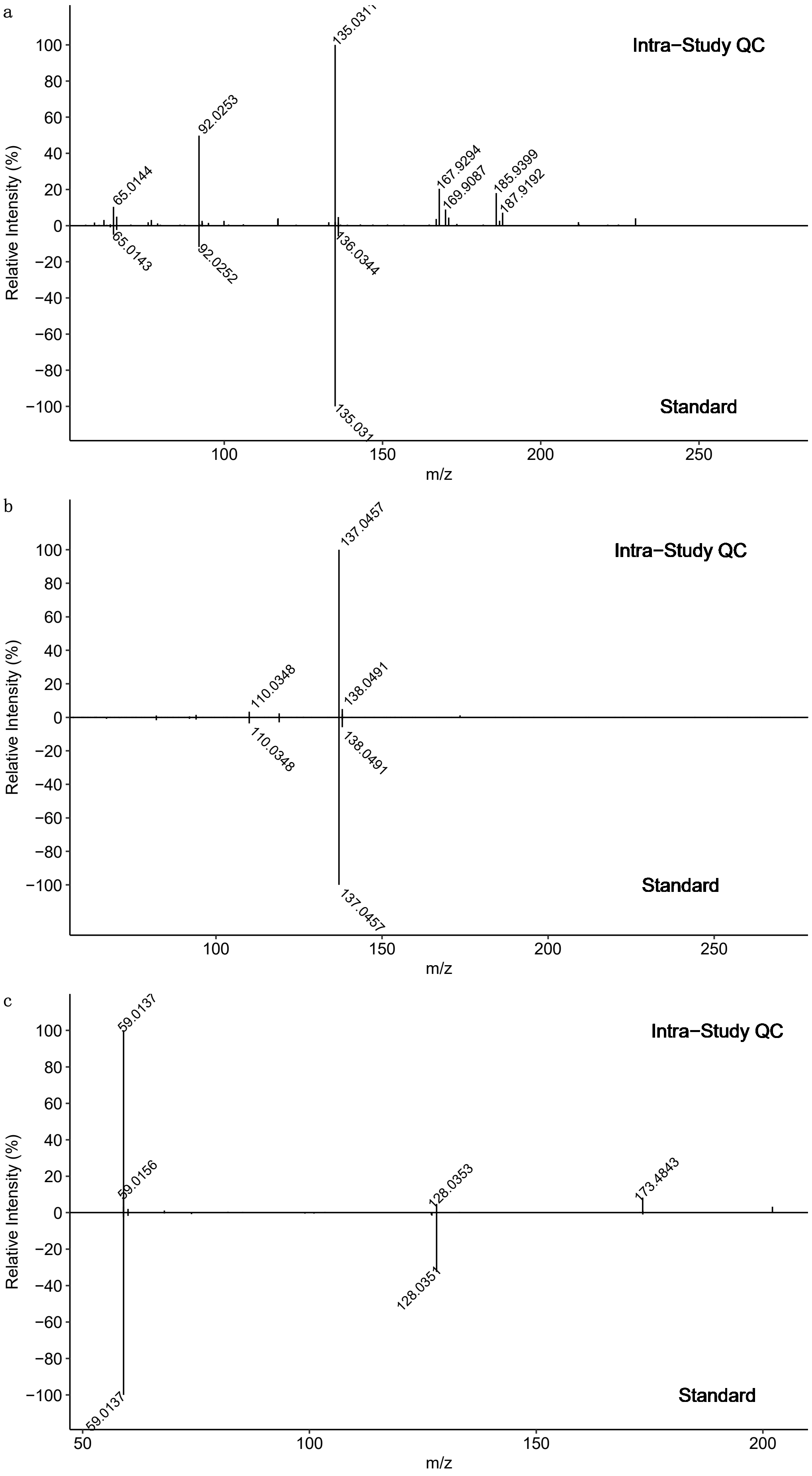
a
Intra−Study QC
Standard
Relative Intensity (%)
m/z
135.031
92.0253
65.0144
167.9294
169.9087
185.9399
187.9192
65.0143
92.0252
136.0344
135.031
b
Intra−Study QC
Standard
Relative Intensity (%)
m/z
137.0457
110.0348
138.0491
110.0348
138.0491
137.0457
c
Intra−Study QC
Standard
Relative Intensity (%)
m/z
59.0137
59.0156
128.0353
173.4843
128.0351
59.0137

Figure S11. Comparison of average (across scans) raw MS/MS spectra of **a** hypoxanthine (precursor ion: *m/z* 271.0697, retention time: 2.3 – 2.8 min), **b** inosine (precursor ion: *m/z* 269.0880, retention time: 4.7 - 5.5 min) and **c** L-glutamine (precursor ion: *m/z* 127.0513, retention time: 7.7 - 8.9 min), measured in an intra-study QC sample (top plot, labelled “intra-study QC”), and the corresponding reference standard (bottom plot, labelled “standard”). The spectral similarity (cosine dot product) scores are 0.91, 1.00 and 0.95 for hypoxanthine, inosine and L-glutamine, respectively.

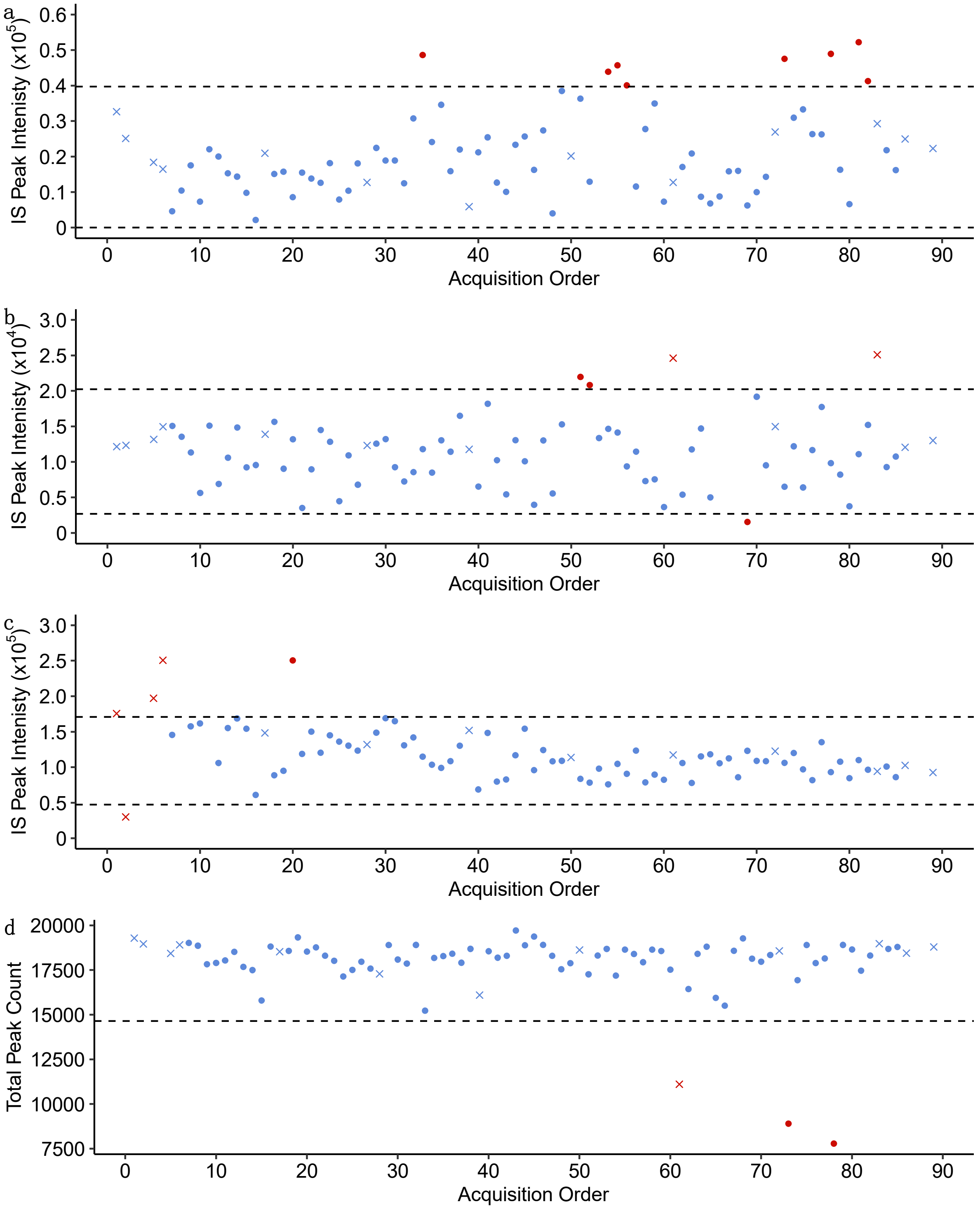

Figure S12. Peak intensity of internal standard, **a** L-tryptophan-indole-d5 $[M+Na]^+$, **b** L-tryptophan-indole-d5 $[M+Na-2H]^-$ and **c** dodecylphosphorylcholine-d38 $[M+Na]^+$, in biological samples (●) and intra-study quality control samples (×) across analytical sequences as measured by polar metabolomics in positive and negative ionisation, and lipidomics in positive ionisation, respectively. Dotted line shows threshold for sample and/or intra-study QC removal, derived from median ± 2.97*MAD (median absolute deviation) of peak intensity across all injections. Accepted and rejected QCs/samples are coloured blue and red, respectively. **d** In the absence of internal standard peaks in the negative ionisation lipidomics data, poor quality samples were removed based on total peak count after spectral stitching. Here the threshold was derived at 80% median peak count across all injections.

Table S7: Ion source parameters for data acquisition by UHPLC-MS(/MS)

Assay	Sheath Gas (Arb)	Aux gas (Arb)	Sweep gas (Arb)	Spray voltage (kV)	Ion transfer tube temperature (°C)	Vaporizer temperature (°C)
HILIC_POS	40	8	1	3.2	200	300
HILIC_NEG	40	8	1	2.7	200	300
